# The enhancement of public real-estate assets through participation and social innovation: Empirical data from Italy

**DOI:** 10.1016/j.dib.2018.09.112

**Published:** 2018-10-03

**Authors:** Alessia Mangialardo, Ezio Micelli

**Affiliations:** Università IUAV di Venezia, Dorsoduro 2206, I-30123 Venice, Italy

**Keywords:** Social innovation, Social capital, Public real-estate property, Bottom-up processes, Urban reuse, Grass-roots participation

## Abstract

For years the enhancement of public real estate assets has been at the center of a lively debate in Italy. Over the last few years, as a consequence of a significant decline of the real estate market, public policies have associated the enhancement of public assets with social innovation. Abandoned buildings without any interest for the real estate market have turned into opportunities for development for profit and non-profit entrepreneurs operating in the most diverse domains, with particular reference to cultural-based activities, digital manufacturing and subsidiary welfare.

The data collected, relating to 50 significant successful experiences throughout Italy, are aimed at highlighting the conditions that favor the enhancement of public property assets in support of social innovation. In particular, the research aims to describe this phenomenon through quantitative data and it offers an effective interpretation of the main characteristics of bottom-up processes in Italy.

Data can be gathered in three groups. The first includes the variables that indicate the location of the case study (region, city, part of the city in which the building is located) and specify the endowment of social capital of the area according to public statistic sources. The second includes the variables related to the dimension (variable considered for size classes) and to the quality of the assets (in particular according to their obsolescence). Finally, the third one considers the juridical and managerial aspects of the relationship between public landlords and self-organized associations and groups (nature of the contract, commitments of the parties, internal organization of associations and groups).

**Specifications table**

**Table:** 

Subject area	*Urban studies; real-estate development*
More specific subject area	*Enhancement of public real-estate properties through social innovation*
Type of data	*Table*
How data was acquired	*Surveys, desk research and official data*
Data format	*Analyzed*
Experimental factors	*The sample includes data collected throughout the national territory and it is representative of almost Italian social innovation initiatives that enhanced abandoned public real-estate properties*
Experimental features	*Firstly, descriptive statistics were provided, in order to briefly describe data. Then a multivariate cluster analysis was performed in order to classify the great heterogeneity of the case studies*
Data source location	*Italy*
Data accessibility	https://www.dropbox.com/s/rgx8d2dqh38ys5i/Specification%20table.xlsx?dl=0
Related research article	*Mangialardo A., Micelli E. (2017) From sources of financial values into commons: Emerging policies for enhancing public real-estate assets in Italy. Paper in Regional sciences.*https://doi.org/10.1111/pirs.12310[6]

**Value of the data**

• The dataset provides the main characteristics of the enhancement of public real-estate assets that have taken place thanks to social mobilization and participation processes in Italy. Today, this is the sole database available on this topic in Italy. In particular, this database provides national quantitative data, making this research original with respect to the topic that is widely debated in Italy from a qualitative point of view.
• The research intersects more detailed variables related to each building and variables that analyze the territory more generally. The combination of variables at different scales makes it possible to explain the principle that originates the start of similar practices in completely different social and economic contexts.
• The data can be used in the most diverse investigations related to the enhancement of public real estate assets and social innovation processes through any multivariate statistics tool.
• The dataset is useful for a wide variety of researches related to emerging urban issues with particular concern to urban regeneration, social mobilization and social innovation processes, and to innovative enhancement policies of public real estate assets.

## 1 Data

The research aims to identify some common patterns that can represent as many predictors for the success of social innovation initiatives. In particular, the goal of the research consists to study how, and to what extent, both the social capital and the promotion of specific public policies are able to determinate a successful outcome of social innovation processes. In this regard, a dataset was designed to verify the cause-effect relationship between some variables that analyze completely different aspects [1], [2], [3], [4], [5], [6], [7], [8].

Variables examined can be resumed in three macro-categories. The first investigates the location of the case study and connects these variables with two topics related to the social innovation initiatives: the endowment of social capital in the territory and the presence of specific public policies to promote these initiatives. Founded on the richness of the city׳s social capital, bottom-up mobilization can radically transform public property into resources that the community can use to promote innovation in both profit and non-profit sectors [8], [8], [9], [10], [11]. Furthermore, the debate on public property enhancement has often – and not merely coincidentally – intersected with that on youth policies. Administrations can promote specific public policies to foster and to encourage the local citizenry to starts social innovation processes [12], [13], [14], [15], [16], [17], [18].

The second group of variables concerns the architectural dimension and the quality of the obsolescence of the assets. Finally, the third one considers the legal and managerial aspects of the connection between public landlords and the users of the assets: the need for flexibility is guaranteed by specific legal relationship between the administration and private parties [5], [9].

## 2 Experimental design, materials, and methods

The dataset with fifty case studies includes all most relevant bottom-up enhancement public real estate initiatives in Italy. Case studies have been selected in Italy. The aim of this dataset is to offer a complete national picture of bottom up processes. The variables of each case study have been acquired through interviews, desk research and field surveys.

The variables have been collected are: regional location, the endowment of social capital of the territory, the city size, location and accessibility of the asset, its size, the resources employed for the property׳s redevelopment, the terms of the contractual arrangement between the public administration and the users of the asset, the managerial model and, finally, the presence or the absence of specific public policies to encourage these process (see Table 1).

**Table 1:** Scale of measure and coding system of the variables.

Clusters	Variables	Scale of measure	Coding system
Location at regional level	North	Nominal	Number
	Centre		
	South		
Location at urban level	Large-sized cities	Ordinal	Number
	Small-sized cities		
Urban location	Central	Ordinal	Number
	Semi-central		
	Suburban		
	Extra-urban		
Type of financing	Self-financing	Nominal	Number
	External financing		
	Both		
Dimensions of the building	Little size	Ordinal	Number
	Medium size		
	Large size		
Presence of social capital	National average	Ratio (continuum)	Percentage
Presence of specific public policies	Yes	Dichotomous	Number
	No		
Type of contract	Bailment	Nominal	Number
	Lease		
	No contract		
Managerial solution	Sole administrator	Nominal	Number
	Consortium		
	Direct assignment		

The regional location interests the geographical position of the asset and it is classified in north, center, and south of Italy. The city size identifies whether the enhancement process started in a city of big sizes or in a small one (less than 50,000 inhabitants). The position indicates the level of centrality of the asset in the city: center, semi-center, periphery, or extra-urban.

The variable concerning the architectural typology concerns the type of the enhanced asset: barrack, theatre, school, industrial building are the main enhanced buildings considered in the survey.

Downing in detail, the variable size groups buildings together by their size: small for buildings characterized by a surface area up to 5000 sq.m, average between 5000 and 20,000 sq.m and large from 20,000 sq.m and above.

The variable “legal terms” indicates the type of agreement between public administration that own the asset and users. There are three possibilities: lease, bailment at no charge, and, when the property was occupied without any authorization, no contractual arrangement.

The variable regarding the funding indicates the economic resources employed to restore the building: funds provided by users, or other resources.

The variable concerning managerial solution represents the management model used to enhance the property. Three possibilities were recognized: "sole administrator" when a single party or a single person administers the regeneration project; "consortium," when more organizations coordinate all the activities in the building and “direct assignment," when the public administration, owner of the asset, directly assigns the spaces of the building to the final users.

The variable concerning the presence of specific public policies explains whether the administration dedicated policies at the local level to encourage social innovation projects.

Finally, the social capital of the territory, that means the endowment in the territory of a local citizenry with a strong civic sense which wishes to participate actively in the development of a territory, was indicated [5], [14], [19], [20], [21]. ISTAT surveys transformed the concept of social capital into an quantitative variable [22].

Most of the variables analysed have been translated into ordinal or nominal scale with the exception of the ISTAT variable, which has been treated in percentage terms by defining the number of case studies above and below the national average (see Table 1).
