# Supplementary material for: The enhancement of public real-estate assets through participation and social innovation: Empirical data from Italy
Source: Data Brief. 2018 Oct 3;21:2379–83. doi: 10.1016/j.dib.2018.09.112 (PMC6280554; doi:10.1016/j.dib.2018.09.112)
Supplement: Supplementary file 1 — Supplementary material [file mmc1.pdf]

Authors have participated in the analysis and interpretation of the data, approving it for the final version.

The paper that authors submitted to this journal is original and it was not been published elsewhere.

The authors have no affiliation or a direct or indirect financial interest with any organization in the subject matter discussed in the manuscript.

Alessia Mangialardo and Ezio Micelli
